# The interplay between COX-2, chemotherapeutic drugs, and chemoresistance in colon cancer

**DOI:** 10.1038/s41598-025-98451-9

**Published:** 2025-05-06

**Authors:** Sally M. Shalaby, Salma A. Shawky, Hassan Ashour, Walaa Sarhan

**Affiliations:** 1 Medical Biochemistry Department, Faculty of Medicine, Zagazig University, Zagazig, Egypt; 2 Surgery Department, Faculty of Medicine, Zagazig University, Zagazig, Egypt

**Keywords:** Colon cancer, COX-2, Chemoresistance, Chemotherapy, Biochemistry, Cancer, Genetics, Biomarkers, Oncology

## Abstract

Chemoresistance and tumor relapse remain major clinical problems. Evidence indicates that COX2/PGE2/EP axis has a critical role in tumorogenesis and chemoresistance. This study assessed the relation of the COX-2 gene expression with chemoresistance in colon cancer (CC) patients. Also, it explored the effect of chemotherapy on COX-2 expression. The study included 24 patients with CC without chemotherapeutic treatment and 24 chemoresistant CC patients. Tumor and adjacent non-neoplastic colon tissue samples were collected and COX-2 mRNA expression was measured. Also, COX-2 and its related genes; TROP2 and DUSP4 expression were analysed in 5 flurouracil and Oxalliplatin treated Caco-2 and SW-620 cells. The results indicated significant upregulation of COX-2 expression in tissues of chemoresistant CC patients when compared with that in CC tissues without chemotherapy (*p* < 0.001). There was a relation between COX-2 expression with lymph nodes, metastases and staging in both groups. Concerning in-vitro experiments, there was a dose dependent significant increase of COX-2, TROP2 and DUSP4 mRNA and protein expression levels in 5flurouracil and Oxalliplatin treated cells. These findings demonstrated that overexpression of COX-2 in the chemoresistant CC patients. Both 5 flurouracil and Oxalliplatin induced COX-2 overexpression and in turn COX-2 upregulation may decrease the response of cancer to chemotherapy.

**Supplementary Information:**

The online version contains supplementary material available at 10.1038/s41598-025-98451-9.

## Introduction

Colorectal cancer (CRC) is the 3rd most common cancer and the 2nd major cause of cancer-related mortality worldwide, with about 900,000 mortalities annually^1^. Even after cancer resection, there was a relapse in 40–50% of cases who died due to metastasis, with an overall 5-year survival < 60%^2^.

Cellular proliferation has a crucial role in tumorigenesis and cyclooxygenase-2 (COX-2) enzyme regulates such process. It plays a role in various biological processes including cellular proliferation, angiogenesis, immunologic functions and inflammatory responses. These processes are vital for tumorogenesis (i.e. initiation and/or progression)^3^. COX-2 expression was predominantly localized in tumour cells, whereas some stromal cells, endothelial cells, and colonic normal mucosa adjacent to the cancer tissue stained weakly^4,5^.

COX-2 overexpression has a role in many precancerous and cancerous lesions of epithelial origin, particularly in the GIT. A cancerous lesion with high COX-2 level showed more aggressiveness with a significant decrease in survival^6^. COX-2 overexpression may decrease cancer’s response to chemotherapy^7^. At the same time chemotherapy may induce the expression of COX-2^8^. Few studies evaluated the crosslink between COX-2, chemotherapy and chemoresistance.

Chemotherapy is mainstay of cancer therapy. Along with debulking the malignant mass via killing tumor cells, chemotherapy also promotes tumor control through immunostimulation^9^. However, chemoresistance and tumor relapse are still significant issues, suggesting that the chemotherapy-immunostimulation is usually not potent, not durable and/or overcome by immune evasion^10^.

Recent report confirmed that cancer cell-intrinsic activation of COX-2 / PGE2 pathway post-chemotherapy altered the inflammatory signaling pathways of treated malignant cells. Furthermore, COX-2 overexpression postchemotherapy impaired the efficacy of PD-1 inhibitors and chemotherapy combinations^11^. Previous studies had implicated PGE2; the major product of COX-2; produced by dying tumor cells postchemotherapy in stimulating the proliferation of tumour cells and tumour repopulation^12,13^.

We hypothesized chemotherapy could upregulate COX-2 in colon cancer (CC) cells and in turn COX-2 upregulation limits the efficacy of chemotherapy. This study assessed COX-2 gene expression and its relation with chemoresistance in colon cancer patients. The study also explored the effect of chemotherapy on COX-2 expression in CC.

## Subjects and methods

### Sample collection

A total of 48 cases diagnosed with CC were included in our study. Twenty four patients with colon cancer who underwent surgical resection before chemotherapeutic or immunotherapeutic treatment and 24 chemoresistant colon cancer patients were included. Tumor and adjacent non-neoplastic colon tissue samples were collected. All specimens were subjected to histopathologic examination before immediate snap-freezing in liquid nitrogen and kept at -80 °C till further analysis. Stage was assessed by American Joint Commission on Cancer guidelines.

Patients with history of malignant tumors in other organs or benign conditions that may be confused with cancer diagnosis were excluded. Written consents were obtained from the participants. The study obtained its approval from the research ethical committee (no 9865 Zu), Faculty of Medicine, Zagazig University. The clinicopatholoical characteristics of the patients are shown in Table 1.

**Table 1:** Clinicopathological characteristics of studied groups.

	Colon cancer without chemotherapy *n*.24	Colon cancer with chemo-resistant *n*.24	χ^2^	*p*
Age year				
Mean ±SD	52.17±10.85	51.37±9.9	0.27*	0.79
Sex				
Male	15 (65.5)	15 (65.5)	0.09	0.77
Female	9 (37.5)	9 (37.5)		
Family history				
Positive	3 (12.5)	5 (20.8)	0.15	0.69
Negative	21 (87.5)	19 (79.2)		
Site				
Right side	12 (50.0)	14 (58.3)	0.336	0.56
Left side	12 (50.0)	10 (41.7)		
Lymph node				
Positive	10 (41.7)	17 (70.8)	4.15	0.042
Negative	14 (58.3)	7 (29.2)		
Metastasis				
Positive	2 (8.3)	5 (20.8)	0.67	0.41
Negative	22 (91.7)	19 (79.2)		
Staging				
II	15 (62.5)	3 (12.5)	12.80	0.002
III	7 (29.2)	16 (66.7)		
IV	2 (8.3)	5 (20.8)		
Histopathologic grade				
Well-differentiated	2 (8.3)	2 (8.3)	1.1	0.58
Moderate- differentiated	21 (87.5)	19 (79.2)		
Poorly- differentiated	1 (4.2)	3 (12.5)		
Type				
Adenocarcinoma	21 (87.5)	19 (79.2)	2.1	0.35
Mucinous carcinoma	3 (12.5)	3 (12.5)		
Signet ring cell carcinoma	0	2 (8.3)		
Serum CEA levels				
Mean ± SD			4.88*	< 0.001

*t test.

### Cell culture

The human colorectal cell line Caco-2 and SW-620 were purchased from American Type Culture Collection and were cultured in RPMI supplemented with 10% fetal bovine serum, penicillin 100 U/mL and streptomycin 100 µg/ml (Lonza Bioproducts). Cells were subcultured twice weekly in a humidified atmosphere at 37 °C, 5% CO2 at CO2 incubator (Heraeus, Langenselbold, Germany). Cells were then plated in 96-well plate (3000 cells/well) and 6 well plates (2.5 × 10^4^/ well) in the above mentioned culture medium. The following samples were prepared: Untreated CRC cells, treated cells for 24 h with three oxaplatin concentrations (1, 5, 10 µg/ml) and treated cells for 24 h with three 5flurouracil concentrations (1, 10, 100 µg/ml)^14^. At the end of the experiment, COX-2, DUSP4 and TROP2 gene expressions were measured using qRT-PCR.

### MTT assay

Caco-2 and SW-620 cells were plated in 96-well plates. Untreated and treated cells with chemotherapy were analyzed to assess growth inhibition of the tumour.

In brief, 10 ul of 5 mg/ml MTT were added to the well with incubation in CO2 incubator for 3 h. The media was then removed; the formed formazan salt was solubilized using DMSO. Cells underwent incubation with DMSO at 37 °C for 30 min and absorption was measured at 570 nm.

### RNA extraction

Homogenization of tissue samples was done in TRIZOL Reagent (50–100 mg tissue/ml TRIZOL) using Polytron power homogenizer. Also, extraction of RNA from cultured cells was carried out post-trypsinization.

Total RNA underwent extraction using RNA extraction kit GENEzol™ Reagent, TriRNA Pure Kit, (Geneaid, China) according to manufacturer’s guidelines and quantified using spectrophotometer at 260 nm.

### cDNA synthesis and qRT-PCR

RNA was reverse transcribed using a Maxime RT PreMix kit following manufacturer’s guidelines. qRT-PCR was done in a Rotor-Gene Q 2 plex Real-Time PCR system (Qiagen, Germany) utilizing TopReal sybergreen master mix (Enzynomics, Korea).The expression levels of the studied genes were normalized to β-actin. Table 2 shows the primer sequences for qRT-PCR. The relative expression was calculated using 2–ΔΔct method.

**Table 2:** Primers sequence used in the study.

Gene	Forward	Reverse
COX-2	5’-GTTCCACCCGCAGTACAGAA-3’	5’-AGGGCTTCAGCATAAAGCGT-3’
DUSP4	5’-CTCCTGTGGGACCCCACTACACGAC-3’	5’-ATG TCTCTCCGGGCAGCATGGTAGG-3’
TROP2	5’-TGTCCTGATGTGATATGTCTGAG-3’	5’-GGGTGAGAGTGGGTTGGG-3’
β-actin	5’-CCATGGGGAAGGTGAAGGTC-3’	5’-CTTCCCGTTCTCAGCCATGT-3’

### Western blot analysis

The cells were lysed in RIPA buffer (Beyotime). The protein concentration was measured according to the BCA kit (Boster Biotech Co. Ltd., Wuhan, China) instructions. Samples proteins were separated on a polyacrylamide gel (TGX Stain-Free FastCast Acrylamide Kit) electropheresis with tube voltage of 80–120 V and transferred to a PVDF membrane (Millipore, Burlington, USA) membrane under 100 mV for 45–70 min. Then the reaction was blocked with 5% bovine serum albumin (BSA) at room temperature for 1 h. Then the membranes were incubated with primary antibodies against COX-2 (sc-19999), TROP2 (sc-376181), Dusp4 (sc-1200) (1:1000 dilution; Santa Cruz Biotechnology) and β-actin (SC-58673) (1: 2000 dilution; Santa Cruz Biotechnology)overnight at 4 °C.Then, the membranes were incubated with corresponding secondary antibodies at room temperaturefor 1 h. After being washed by Tris-buffered saline plus Tween (TBST) buffer for 3 times, densitometric analysis of the immunoblot was performed to quantify the amounts of COX2, TROP2 and Dusp4 in all studied samples against control sample β-actin by protein normalization on the ChemiDoc MP imaging system (version 3) produced by Bio-Rad (Hercules, CA).

### Statistical analysis

The SPSS program V 22 was used to analyze the data. Data were described as mean ± SD. Categorical data were described as frequencies and percentages. Student’s t- test, Mann-Whitney, Anova and Kruskall Wallius, chi square, Pearson correlation coefficient were utilized for data analysis. Significance was set at *p* < 0.05.

## Results

### COX-2 gene and its related genes expression in tissue samples of colon cancer patients

COX-2 mRNA expression was significantly higher in tissues of chemoresistant CC cases when compared with that in CC tissues without chemotherapy (*p* < 0.001) or with normal adjacent tissues (*p* < 0.001) (Table 3).

Dual-specificity MAPK phosphatase (DUSP4) and trophoblast cell-surface antigen 2 (TROP2) mRNA expression also studied; the expression levels of both DUSP4 and TROP2 were higher in chemoresistant CC tissues (12.41 ± 2.45, 11.65 ± 3.6 respectively) when compared with CC tissues without chemotherapy (4.32 ± 1.15, 3.25 ± 1.47 respectively) (*p* < 0.001 for both). There were significant positive correlation between COX-2 expression levels and DUSP4 gene expression (*r* = 0.851, *P* < 0.001) and also between COX-2 and TROP2 expression levels (*r* = 0.866, *P* < 0.001) in all colon cancer tissues.

**Table 3:** COX-2 gene expression in tissue samples of the studied groups.

	Adjacent healthy tissue *n*.24	Colon cancer without chemotherapy group *n*.24	Chemo resistant colon cancer group *n*.24	f	*p*
Mean ± SD	1.18 ± 0.79	5.85 ± 1.62*	13.1 ± 2.07*#	336.4	< 0.001
Range	0.98–4.91	4.82–10.78	6.38–15.2		

f = Anova test.

* *p* < 0.001 when compared to normal group.

#*p* < 0.001 when compared to to colon cancer without chemotherapy.

### Relation between COX-2 gene expression and the clinicopathologic characteristics of the studied groups

To study the effect of COX-2 overexpression on colon cancer, we analysed the association between COX-2 expression and the clinicopathological features. We found a significant COX-2 overexpression with lymph node and metastasis in both CC group without chemotherapy (*p* = 0.001, 0.028 respectively) or with chemotherapy group (*p* = 0.005, 0.012 respectively). The expression COX-2 was significantly higher in stage IV than stage II and III in both groups. No significant differences existed between the COX-2 expressions and the tumour type or pathology or site (Table 4).

**Table 4:** Relation between COX-2 gene expression and the clinicopathologic characteristics of studied groups.

	Without chemotherapy group *n*.24	u	*p*	Chemo resistant group *n*.24	u	*p*
Site						
RT colon	6.42 ± 2.22	0.433	0.665	13.03 ± 2.43	0.38	0.70
LT colon	5.28 ± 0.19			13.15 ± 1.89		
Lymph node						
Positive	6.81 ± 2.27	3.25	0.001	13.84 ± 0.74	2.83	0.005
Negative	5.17 ± 0.20			11.30 ± 3.15		
Metastasis						
Yes	10.13 ± 0.93	2.19	0.028	14.48 ± 0.73	2.52	0.012
No	5.46 ± 1.02			12.74 ± 2.18		
Stage						
Stage II	5.21 ± 0.23	7.26	0.02^k^	11.33 ± 3.16	8.07	0.018^k^
Stage III	5.95 ± 1.56			13.68 ± 0.60		
Stage IV	10.37 ± 0.59			14.20 ± 1		
Histopathology						
Well differentiated	5.33 ± 0.15	0.66	0.72^k^	13.38 ± 0.08	1.45	0.26^k^
Moderately differentiated	5.94 ± 1.75			12.78 ± 2.21		
Poorly differentiated	5.2 ± 00			14.94 ± 0.42		
Type						
Adenocarcinoma	5.46 ± 0.95	1.44	0.15	12.78 ± 2.21	1.45	0.263^k^
Mucinous carcinoma	8.64 ± 3.01			14.94 ± 0.42		
Signet ring cell carcinoma	–			13.38 ± 0.08		

u: Mann whitnney u test, K:Kruskall Wallius test, *p* < 0.05: significant.

### The effect of chemotherapy on growth Inhibition of colorectal cells

**Fig. 1:**
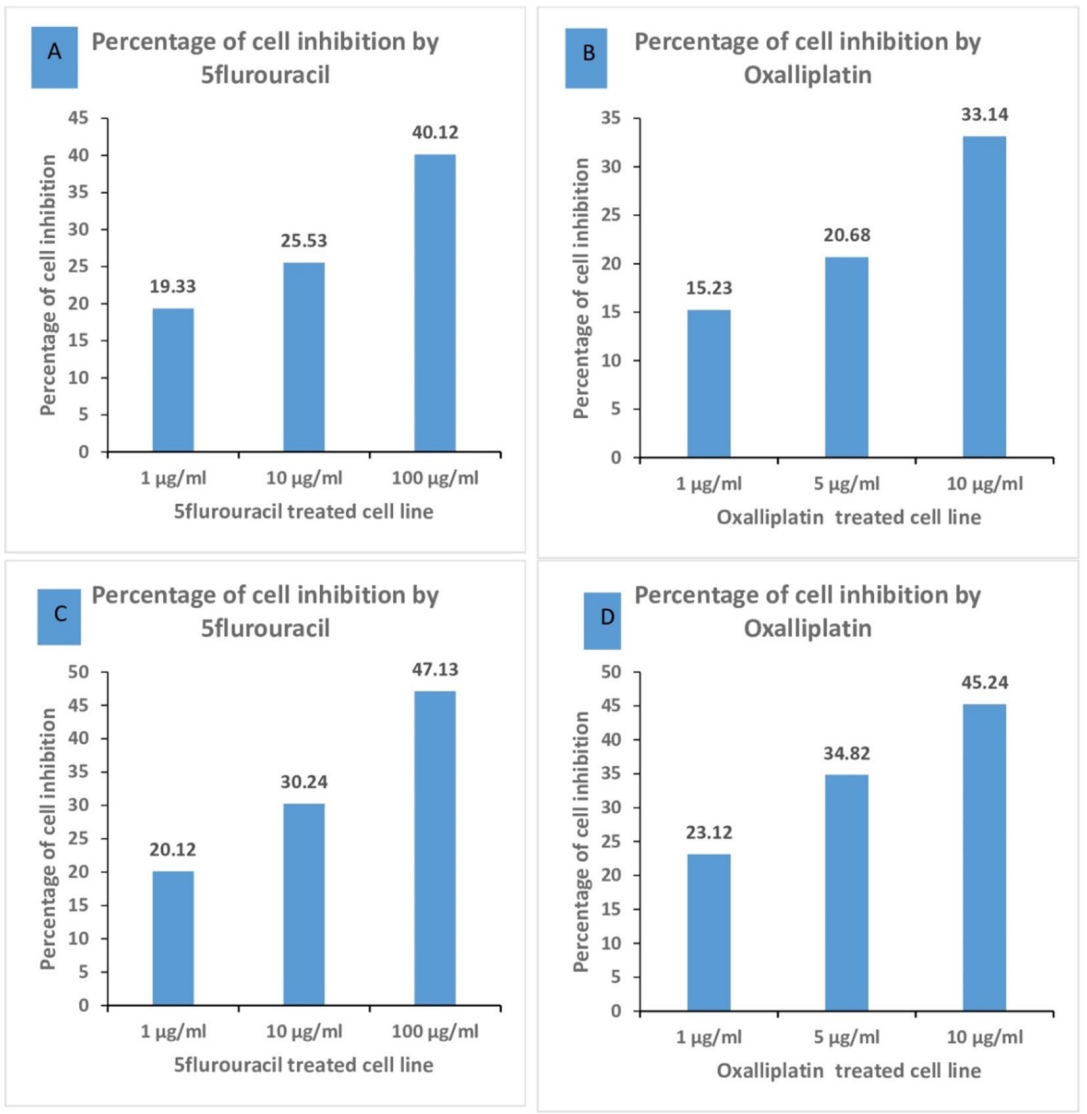
Percentage of cell inhibition in: A, B: Caco-2 cell line C, D: SW-620 cell line (both treated by 5 flurouracil or Oxaliplatin).

Both 5 flurouracil and oxaplatin significantly suppressed, in a dose-dependent manner, the growth of Caco-2 cells (40.12% and 33.14% inhibition at the highest concentration, respectively) and SW-620 cells (47.13% and 45.24% inhibition at the highest concentration, respectively) (Fig. 1).

### COX-2 and its related genes mRNA expression in 5 Flurouracil (1, 10, 100 µg/ml) treated cell line

A dose-dependent significant increase of COX-2 mRNA expression was observed in 5flurouracil treated cell lines (*p* < 0.001). Moreover, a dose-dependent significant increase of DUSP4 mRNA expression was observed in 5flurouracil treated Caco-2 cell line (*p* < 0.001) and SW-620 cell line (*p* < 0.001). A significant upregulation of TROP2 gene expression was observed in the three treated groups when compared to non treated one (Table 5).

**Table 5:** COX-2 and its related genes mRNA expression in 5flurouracil (1, 10, 100 µg/ml) treated cell lines.

Gene expression relative to β-actin	Cell line	No treatment	Chemotherapy dose			f	p
			1 µg/ml	10 µg/ml	100 µg/ml		
COX-2	Caco-2	1.03 ± 0.06	2.3 ± 0.25*	4.09 ± 0.46*^#^	7.4 ± 0.71*^#$^	191.1	< 0.001
	SW-620	1.02 ± 0.07	3.14 ± 0.15*	4.53 ± 0.23*^#^	7.33 ± 0.62*^#$^	301.12	< 0.001
DUSP4	Caco-2	1.01 ± 0.05	2.03 ± 0.57*	3.28 ± 0.21*^#^	5.47 ± 0.89*^#$^	62.5	< 0.001
	SW-620	1.04 ± 0.03	3.56 ± 0.16*	4.34 ± 0.35*^#^	6.18 ± 0.55*^#$^	201.25	< 0.001
TROP2	Caco-2	1.02 ± 0.02	3.35 ± 0.3*	5.07 ± 1.15*	6.7 ± 0.94*^#^	50.3	< 0.001
	SW-620	1.02 ± 0.01	3.91 ± 0.35*	5.77 ± 0.65*^#^	7.89 ± 0.32*^#$^	262.31	< 0.001

Data were expressed mean ±SD. f= Anova test (5 samples in each group).

*p<0.05 when compared to non treated group.

#*p*<0.05 when compared to 1 μg/ml 5Fu treated group.

$*p*<0.05 when compared to 10 μg/ml 5Fu treated group.

### COX-2 and its related genes mRNA expression in Oxaplatin (1, 5, 10 µg/ml) treated cell line

A dose-dependent significant increase of COX-2 mRNA expression (*p* < 0.001) was observed in Oxalliplatin treated Caco-2 and SW-620 cells. Also, a significant increase of DUSP4 mRNA expression was observed in the three treated groups in comparison to non treated one (*p* < 0.001) for both cell line. There was a dose-dependent significant increase of TROP2 gene expression in the three treated groups (Table 6).

**Table 6:** COX-2 and its related genes mRNA expression in Oxalliplatin (1, 5, 10 μg/ml) treated cell lines.

Gene expression relative to β-actin	Cell line	No treatment	Chemotherapy dose			f	p
			1 μg/ml	5 μg/ml	10 μg/ml		
COX-2	Caco-2	1.03 ± 0.13	2.72 ± 0.31*	5.3 ± 0.54*^#^	8.5 ± 1.82*^#$^	57.3	< 0.001
	SW-620	1.02 ± 0.05	3.14 ± 0.53*	6.102 ± 0.34*^#^	8.67 ± 0.57*^#$^		
DUSP4	Caco-2	1.02 ± 0.14	3.45 ± 0.57*	6.19 ± 1.36*^#^	7.6 ± 1.44*^#^	39.4	< 0.001
	SW-620	1.05 ± 0.02	3.9 ± 0.32*	6.27 ± 0.35*^#^	8.15 ± 0.67*^#$^	279.33	< 0.001
TROP2	Caco-2	1.03 ± 0.01	1.49 ± 0.19*	3.4 ± 0.44*^#^	5.4 ± 0.84*^#$^	43.3	< 0.001
	SW-620	1.06 ± 0.04	2.15 ± 0.24*	4.28 ± 0.56*^#^	5.37 ± 0.77*^#$^	79.78	< 0.001

Data were expressed Mean ± SD. f = Anova test (5 samples in each group).

*p < 0.05 when compared with non-treated group.

#*p* < 0.05 when compared with 1 μg/ml Oxalliplatin treated group.

$*p* < 0.05 when compared with 5 μg/ml Oxalliplatin treated group.

### Western blot analysis results

**Fig. 2:**
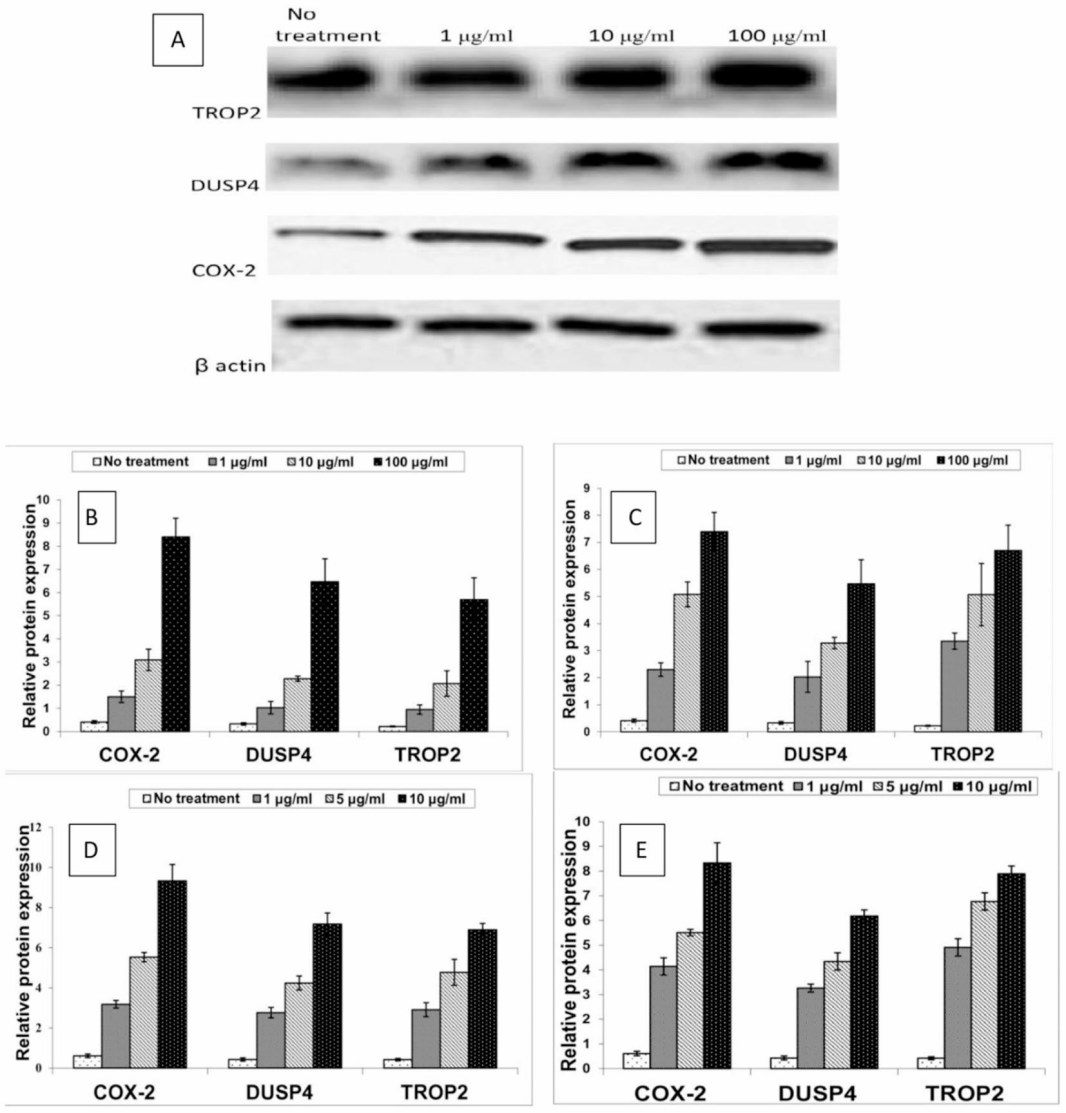
Relative protein levels of COX-2, DUSP4 and TROP2 (A) A representative western blot of COX-2, DUSP4 and TROP2 expression in no treatment, 1, 10, 100 µg/ml 5flurouracil treated Caco-2(full blots available in supplementary figures S1–S4) (B) Relative protein level of COX-2, DUSP4 and TROP2 in 5flurouracil treated Caco-2 (C) Relative protein level of COX-2, DUSP4 and TROP2 in 5flurouracil treated SW-620 (D) Relative protein level of COX-2, DUSP4 and TROP2 in Oxalliplatin treated Caco-2 (E) Relative protein level of COX-2, DUSP4 and TROP2 in Oxalliplatin treated SW-620.

The protein expression levels of COX-2, TROP2 and DUSP4 significantly increased in a dose dependent manner in 5flurouracil treated cell lines in both Coca2 and SW620. Also, they were significantly increased in a dose dependent manner in oxalliplatin treated Caco-2 and SW-620 (Fig. 2).

## Discussion

Most patients affected with CRC have been undergone systemic chemotherapy but its efficacy is often hampered by resistance mechanisms in tumor cells. The efficacy of chemotherapy is correlated with cellular and molecular mediators of anti-tumor immunologic response^10^.

Evidence indicates that COX2/PGE2/EP axis has a critical role in tumorogenesis (initiation and/or progression) and chemoresistance^7^. The present study revealed that COX-2 mRNA expression was significantly higher in chemoresistant CC tissues than in CC tissues without chemotherapy.

The COX2/PGE2/EP axis has a role in almost all stages of tumorogenesis, which include cancer stem cells (CSC) repopulation, epithelial–mesenchymal transition (EMT) progression which all contribute to chemoresistance^7^. Upregulation of COX-2 has a significant association with increased CSC population and stimulates CSC self-renewal and proliferation in colon cancer^15^. In CRC, hypoxaemia could mediate COX-2 upregulation and was associated with increased CSC subpopulation and chemoresistance^16^. The COX2/PGE2/EP axis might also have a role in EMT-related chemoresistance^18^. COX inhibitors could rescue E-cadherin expression and subsequent reversion of EMT process in many cancer cell lines^19^.

Previous study in patients with advanced (stage IV) CRC were treated with fluoropyrimidine found that COX-2 gene showed a significantly lesser expression in responding tumors compared with the nonresponders^20^. Such finding supported the combination of anti-COX-2 agents with conventional chemotherapeutic drugs to enhance its effectiveness. The potential anti-apoptotic effect of COX-2 suggested that tumours with COX-2 overexpression showed less sensitivity to chemotherapeutic drugs that induced apoptosis^21,22^. Also, COX-2 could up-regulate multidrug resistance gene MDR1/pgp70^23^ suggesting another mechanism of chemoresistance promoted by COX-2.

The present study revealed a significant COX-2 overexpression with lymph nodes, metastasis and with advanced stages of colon cancer. No significant differences existed between the COX-2 expressions and the tumour type, pathology or site. In colorectal carcinoma, previous studies found high COX-2 expression was linked to lymph node and distant metastases as well as advanced AJCC stage^24,25^. Kazem et al.^26^ reported a significant relationship between pathologic grade and COX-2 positivity scores in colorectal cancer. Both serum and tissue COX-2 levels had a correlation with T stage, N stage and TNM stage^27^. So, COX-2 was identified as a major metastasis progression gene^28^.

The result of the present study that COX-2 overexpression in CC patients receiving chemotherapy than in CC patients without chemotherapy may lead to another explanation that found in other studies. Mercer et al. studied^29^ the effect of 5 flurouracil (5-FU) or 5-FU combination chemotherapy on COX-2 expression in biopsies from 14 esophageal cancers (obtained pre- and post-chemotherapy) and in tumour-derived cells exposed to 5-FU in vitro from a series of 44 cancers (breast, ovarian, esophageal and colonic cancers). COX-2 showed overexpression after treatment with 5-FU or 5-FU combination chemotherapy in all tumors, whether measured in biopsies obtained pre- and post- chemotherapy (4-folds increase) or in tumour-derived cells treated with 5-FU in vitro (24-folds increase). Recently, Bell et al.^11^ confirmed that COX-2/PGE2 pathway activation by CTX-treated tumour cells occurred regardless of the type of tissue and the type of chemotherapy. Screening a compound library of 1280 approved chemotherapeutic agents found that all drugs enhanced COX-2 expression signifying that COX-2/PGE2 axis activation occurred independent of the mechanism of action of the drug.

In the present study, a significant increase of COX-2 mRNA and protein expression levels was found in both 5flurouracil and oxalliplatin treated Caco-2 and SW-620 cell lines in a dose-dependent manner. This confirmed that chemotherapy could enhance COX-2 upregulation in CC cells. In non-small cell lung cancer cases, Altorki and his team^8^ found that intra-tumoral COX-2 and PGE2 levels were around 3-folds greater in cases that administered pre-operative chemotherapy, compared to cases that underwent surgical resection only. They showed that chemotherapy increased COX-2–related PGE2 production in cancer cells but not in healthy cell. This agreed with the fact that cytotoxic therapy caused a greater injury of tumour tissue as compared to healthy tissue.

We found a significant overexpression of COX-2-related genes; TROP2 and DUSP4 in CC patients receiving chemotherapy. Also, the results showed a dose dependent increase of TROP2 and DUSP4 mRNA expression and their protein levels in both 5flurouracil and oxaliplatin treated Caco-2 and SW-620 cells. Trophoblast cell-surface antigen 2 (TROP2) is a glycoprotein expressed during embryonic and fetal development. TROP2 has a role in cell proliferation, binding, motility, and metastases. TROP2 is consistently induced by COX-2 overexpression^30^. Hidalgo-Estévez et al. confirmed that COX-2 overexpressing colon carcinoma cells present high levels of TROP2^31^. In our study we found a significant correlation between COX-2 and TROP2 mRNA expression in CC tissues. There was also a tendency for Tacstd2 (TROP2) to be upregulated with COX-2 overexpression in CT26 cells, as it happened in HT29 human cells^32^. On the other hand, Cheng et al. displayed that TROP2 overexpression resulted in an evident increase in the levels of COX-2 in nasopharyngeal carcinoma cells^33^. TROP2 can promote drug resistance, an effect decreased by TROP2 inhibitors^34^. In many patients, cytotoxic therapy is associated with TROP2 overexpression. For instance, tamoxifen significantly induced TROP2 overexpression in breast cancer cell line^35^.When HCT-116 human colon cancer cells were exposed to oxaliplatin, TROP2 showed overexpression^36^.

Dual-specificity MAPK phosphatase (DUSP4) also has upregulated by COX-2^31^. The present study found that a significant correlation between COX-2 and DUSP4 mRNA expression in CC tissues. DUSP4 mRNA was overexpressed, in an MEK-dependent manner, in CRC cells^37,38^. The overexpressed DUSP4 in CRC cell line reduced its sensitivity to doxorubicin^39^. DUSP4 gene was reported to be the highest induced in cetuximab-resistant CRC cells^40^.

More trials are needed to validate our results. The involvement and the precise mechanism of COX-2 effects in reducing chemotherapeutic outcome need further evaluation. Finally, the effects of COX-2 inhibitors on the antitumor efficacy of different classes of chemotherapeutic drugs should be evaluated. So a protocol for successful use of COX-2 inhibitors in clinical applications to CRC could be recommended.

In conclusions, the findings demonstrated that COX-2 overexpression in the chemoresistant CC. Both 5 flurouracil and oxalliplatin induced COX-2 overexpression and in turn COX-2 upregulation could decrease the response of cancer cells to chemotherapy.

## Electronic supplementary material

Below is the link to the electronic supplementary material.

Supplementary Material 1

## Data Availability

The data that support the findings of this study are not openly available and are available from the corresponding author upon reasonable request.
