# Supplementary material for: The interplay between COX-2, chemotherapeutic drugs, and chemoresistance in colon cancer
Source: Sci Rep. 2025 May 6;15:15837. doi: 10.1038/s41598-025-98451-9 (PMC12056169; doi:10.1038/s41598-025-98451-9)
Supplement: Supplementary file 1 — Supplementary Material 1 [file 41598_2025_98451_MOESM1_ESM.docx]

**Supplementary information**


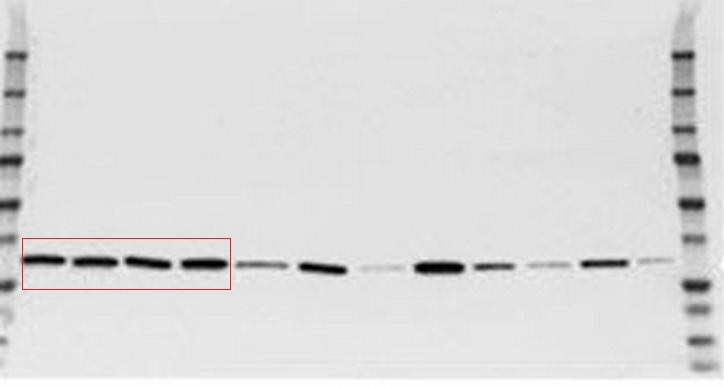


45KDa

**Figure S1:** Uncropped version of β-actin western blot. Protein expression of β-actin in cell line determined by western blotting


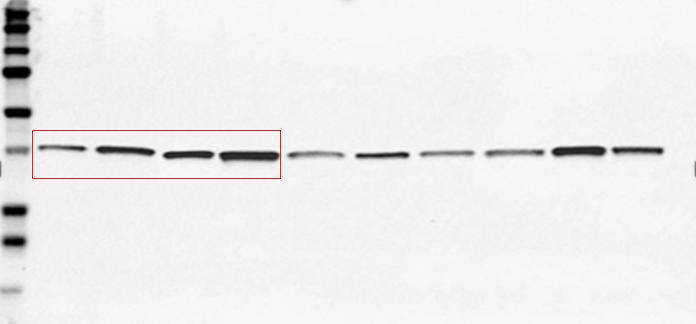


70KDa

**Figure S2:** Uncropped version of COX-2 western blot. Protein expression of COX-2 in cell line determined by western blotting


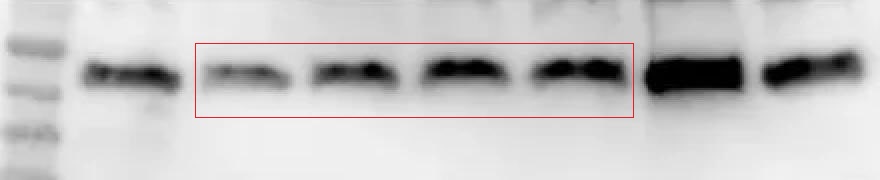


35 KDa

**Figure S3:** Uncropped version of DUSP4 western blot. Protein expression of DUSP4 in cell line determined by western blotting


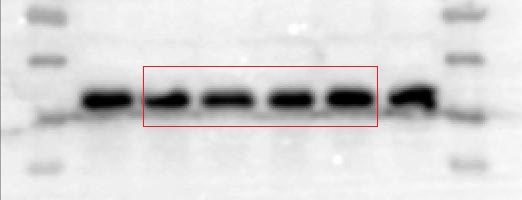


32 KDa

**Figure S4:** Uncropped version of TROP2 western blot. Protein expression of TROP2 in cell line determined by western blotting
